# Case Report: The hybrid cable technique in transcatheter mitral valve implantation with Myval THV—Technical insights from three high-risk cases

**DOI:** 10.3389/fcvm.2026.1682730

**Published:** 2026-02-10

**Authors:** Giuseppe Nasso, Giovanni Goffredo, Antonio Pignatelli, Alfredo Marchese, Flavio Fiore, Antongiulio Valenzano, Giacomo Errico, Giacomo Schinco, Raffaele Bonifazi, Tommaso Loizzo, Dritan Hila, Walter Vignaroli, Giuseppe Speziale, Gaetano Contegiacomo

**Affiliations:** 1 Department of Cardiac Surgery, Anthea Hospital and Santa Maria Hospital GVM Care&Research, Bari, Italy; 2 Department of Medicine and Surgery, LUM University, Casamassima/Bari, Italy; 3 Department of Cardiac Surgery, San Carlo di Nancy Hospital GVM Care & Research, Rome, Italy; 4 Department of Health and Life Sciences, European University of Rome, Rome, Italy

**Keywords:** mitral, mitral valve, Myval, Myval transcatheter heart valve, transcatheter

## Abstract

**Background:**

Transcatheter mitral valve implantation (TMVI) is increasingly utilized as a minimally invasive alternative for high-risk or inoperable patients with degenerated mitral prostheses or extensive mitral annular calcification (MAC). Despite its growing adoption, the procedure remains technically challenging due to complex mitral anatomy, difficulties in achieving coaxial alignment, and risks such as left ventricular outflow tract (LVOT) obstruction. The transfemoral-transseptal route, while less invasive, often provides suboptimal control and stability. Conversely, transapical access offers enhanced alignment but at the cost of increased surgical trauma. There is an unmet need for techniques that balance precision with minimal invasiveness.

**Objectives:**

The aim of this work was to describe the technical rationale, procedural methodology, and early clinical outcomes of a novel hybrid cable-assisted TMVI approach using the Myval transcatheter heart valve (THV). This technique is designed to optimize device coaxiality and control while minimizing apical trauma.

**Methods:**

We retrospectively reviewed three high-risk patients treated with a cable-assisted TMVI approach between October 2024 and May 2025. The technique involved the creation of a through-and-through rail between the right femoral vein and the left ventricular apex. The apical site was used exclusively for guidewire externalization, while the prosthesis was delivered entirely via the transfemoral route. Real-time transesophageal echocardiography and fluoroscopy guided the procedure. Patients were selected based on unfavorable anatomy for standard transseptal access and deemed high risk by a multidisciplinary heart team.

**Results:**

All procedures were technically successful without surgical conversion or cardiopulmonary bypass. Patients included one woman and two men (mean age 71.7 years; range 65–78), with indications of bioprosthetic degeneration (*n* = 2) and MAC (*n* = 1). All were New York Heart Association (NYHA) class III–IV preoperatively. Procedural outcomes showed acceptable post-deployment gradients (4.5–5.8 mmHg), no paravalvular leak, and no LVOT obstruction. One patient required repositioning of a misaligned valve using the stabilized rail, avoiding hemodynamic compromise. No embolization or peri-procedural stroke occurred. All patients were discharged in NYHA class I–II.

**Conclusions:**

This report presents the first dedicated case series of TMVI using a cable-assisted hybrid rail strategy with the Myval THV. The technique enabled precise and stable valve deployment in anatomically complex settings while minimizing myocardial trauma. Early outcomes suggest that it is a feasible, safe, and reproducible option for patients at high surgical risk or with contraindications to conventional TMVI routes. These preliminary findings support further investigation in broader clinical cohorts.

## Introduction

Transcatheter mitral valve implantation (TMVI) has emerged as a life-saving therapeutic alternative for patients with degenerated mitral bioprostheses, failed surgical repair, or extensive mitral annular calcification (MAC) who are deemed inoperable or high risk for reoperative surgery. Recent registry data have demonstrated encouraging outcomes in this high-risk population, with procedural success rates approaching 90% in experienced centers and an increasingly favorable safety profile when proper patient selection and pre-procedural imaging are applied (1, 2). As TMVI expands beyond the aortic field, the anatomical and technical challenges inherent to the mitral position—such as the risk of left ventricular outflow tract (LVOT) obstruction, unpredictable annular geometry, and the need for precise coaxial alignment—have become increasingly apparent. Among these, LVOT obstruction remains the most feared complication, with incidence rates up to 15% in valve-in-MAC (ViMAC) cases, particularly when the anterior mitral leaflet is not resected or displaced (3, 4).

The transfemoral transseptal approach is currently the most widely adopted route due to its percutaneous nature and favorable recovery profile. Nevertheless, this route often results in poor coaxiality and limited stability in anatomically distorted settings, particularly in cases of ViMAC, previous mitral rings, or horizontal mitral-aortic angulation. In addition, the long and tortuous delivery path may reduce the precision of device deployment, especially in the setting of annular calcification or prior surgical rings, where resistance and malalignment are common. Conversely, the transapical (TA) approach allows for superior device control and alignment but is associated with greater surgical invasiveness, pleural complications, and the need for large introducer systems (5). While the transapical route has been pivotal in early TMVI experience, particularly with devices such as Tendyne, it remains limited by its impact on myocardial integrity and post-procedural recovery.

To address this access dilemma, hybrid strategies have been proposed that aim to combine the precision of transapical control with the minimalism of transfemoral delivery. Among these, the cable-assisted or “through-and-through rail” approach has been explored with other transcatheter valves, such as SAPIEN or Tendyne, in isolated case reports (6). This approach creates a stabilized delivery path by exteriorizing a guidewire from the femoral vein through the left ventricular apex, improving alignment and reducing the risk of device displacement during deployment. However, its application in conjunction with the Myval transcatheter heart valve (THV) has not been previously described in a clinical case series.

The Myval THV (Meril Life Sciences, India) offers several properties that may favor its off-label use in the mitral position: a balloon-expandable design, intermediate sizing options, a low-profile delivery system, and favorable radial strength (7).

These characteristics allow for better adaptability to diverse anatomical configurations and contribute to controlled expansion and secure anchoring in previously implanted surgical prostheses or annuloplasty rings (8). Recent experiences have demonstrated its utility in valve-in-valve and valve-in-ring TMVI procedures, with acceptable gradients and good procedural outcomes, comparable to those observed with more established balloon-expandable devices such as SAPIEN 3 (2). Nevertheless, in complex anatomies requiring enhanced delivery stability and precise deployment, further refinements in technique may be necessary to maximize its potential.

In this context, we adopted a cable-assisted hybrid approach that establishes a through-and-through guidewire rail between a right femoral venous access and a minimal transapical surgical exposure. Crucially, the prosthesis is delivered entirely through the femoral route, while the transapical entry site serves only for guidewire externalization—eliminating the need for large-caliber sheaths or prosthesis passage through the ventricular wall. This configuration draws inspiration from similar techniques employed in aortic interventions and is tailored to improve directional control and coaxiality without incurring the full burden of transapical access (5, 6).

This configuration preserves coaxiality and directional control while minimizing myocardial trauma, procedural time, and bleeding risk. It may prove particularly useful in anatomies where standard transseptal navigation is high risk, or where annular orientation and prosthetic angles make stable delivery challenging. In addition, by leveraging the lower profile and sizing flexibility of the Myval system, this approach may overcome some of the limitations posed by rigid or calcified mitral annuli, offering a versatile and less invasive alternative for select high-risk patients.

In this study, we present the technical rationale and early clinical results of three high-risk patients treated with the cable-assisted TMVI technique using the Myval THV. To our knowledge, this represents the first dedicated report of this hybrid rail strategy applied to the Myval system in the mitral position, highlighting its potential to expand the feasibility of TMVI in complex anatomical scenarios.

## Materials and methods

### Study design and setting

We conducted a retrospective case series of three consecutive high-risk patients who underwent transcatheter mitral valve implantation (TMVI) using a hybrid cable-assisted approach with the Myval transcatheter heart valve (Meril Life Sciences, India). All procedures were performed between October 2024 and May 2025 at a single tertiary cardiac surgery center with an integrated heart team comprising interventional cardiologists, cardiac surgeons, anesthesiologists, and echocardiographers. The study was approved by the institutional ethics board, and written informed consent was obtained from all participants.

### Patient selection

Patients were eligible for the cable-assisted TMVI approach based on the following criteria:

High or prohibitive surgical risk (EuroSCORE II ≥8%);
Degenerated surgical mitral bioprosthesis or severe mitral annular calcification (MAC); and
Anatomic features deemed unsuitable for isolated transseptal delivery, such as hostile annular angles, severe mitro-aortic angulation, or distorted prosthetic orientation.

Each case was discussed and approved by the institutional multidisciplinary heart team. Preoperative evaluation included transthoracic and transesophageal echocardiography, contrast-enhanced cardiac CT for annular sizing and landing zone evaluation, prediction of LVOT obstruction after TMVR, and full laboratory and functional assessment.

### The important role of cardiac computed tomography

Computed tomography (CT) is essential for comprehensive anatomical assessment and risk stratification in candidates for percutaneous mitral valve replacement with a hybrid transseptal-transapical cardiac access. Detailed CT analysis enables accurate measurement of mitral annular dimensions—including anteroposterior and intercommissural diameters, annular area, perimeter, and three-dimensional saddle-shaped geometry—which are crucial for appropriate device sizing and anchoring. The extent, distribution, and asymmetry of mitral annular calcification are carefully evaluated, as they influence prosthesis stability and the risk of paravalvular leak (PVL) or annular injury. A critical determinant of access strategy is the angulation of the mitral annular plane relative to the left ventricular long axis, the interatrial septum, and the aortic annulus. Marked annular angulation or horizontalization of the mitral valve may hinder coaxial alignment and controlled deployment through a purely transseptal approach, thereby favoring the addition of a transapical access to improve device trajectory and stability. CT-based simulation is also used to estimate the predicted neo-left ventricular outflow tract (neo-LVOT) area after valve implantation, integrating annular dimensions, anterior mitral leaflet length, and ventricular geometry. A critically reduced neo-LVOT represents a major contraindication or requires alternative access and procedural modifications. Furthermore, CT evaluation of left ventricular size, apical wall thickness, trabeculation, and the annulus-to-apex distance is fundamental to assess the feasibility and safety of transapical puncture, ensuring optimal control during valve delivery while minimizing the risk of ventricular injury and LVOT obstruction.

### Predicting LVOT obstruction in transcatheter mitral valve implantation

**Figure 1:**
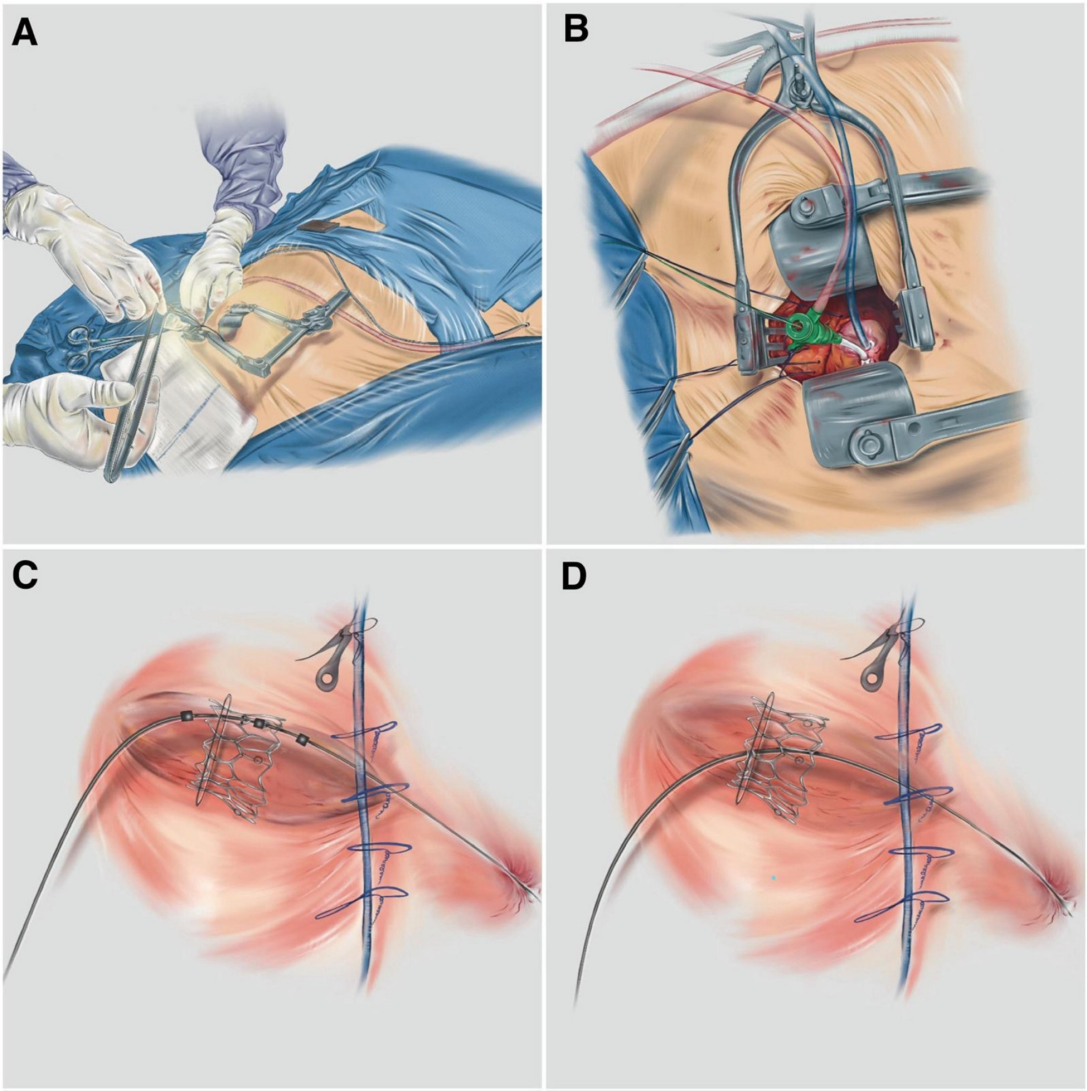
**(A)** Exposure and preparation of the apical and femoral accesses; **(B)** the small-gauge apical introducer into the LV cavity; **(C)** the deployment of the balloon-expandable THV; and **(D)** the final position of the ViV TMVI (patient 2). Illustrations by Debora Gregorio.

**Figure 2:**
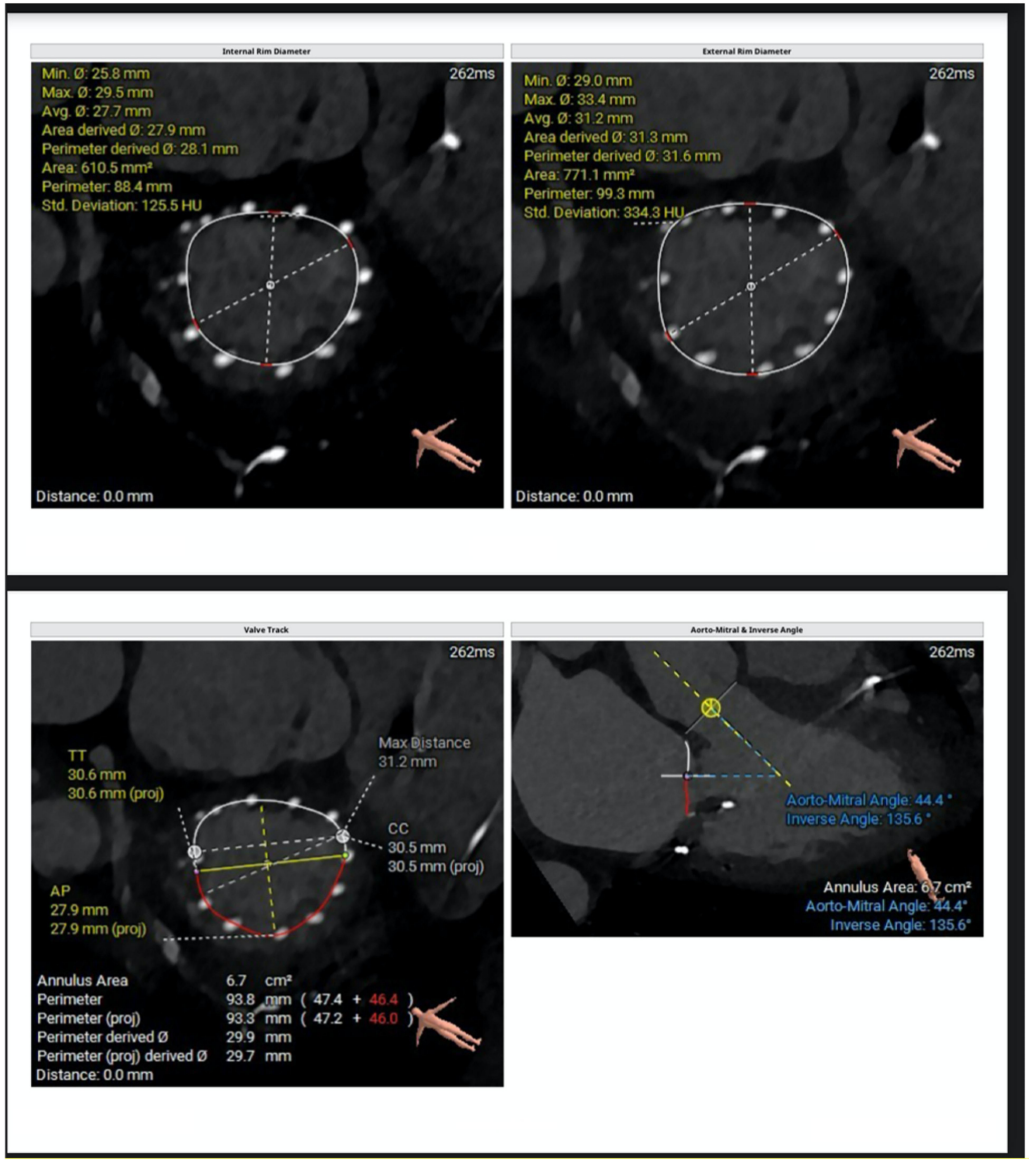
CT-derived measurements include mitral annular geometry with internal and external rim diameters, annular area and perimeter, and anteroposterior and commissure-to-commissure dimensions. The valve tracking analysis illustrates the spatial relationship between the mitral annulus and the left ventricular cavity. Additionally, the aorto-mitral angle and inverse angle are quantified to evaluate annular orientation and the potential impact on device alignment and left ventricular outflow tract interaction.

During transcatheter mitral valve interventions, an elongated neo-left ventricular outflow tract (neo-LVOT) may be created within the left ventricle, whereas the native LVOT—bounded by the basal septum and the intervalvular fibrosa—remains unaltered. The neo-LVOT arises from posterior displacement of the anterior mitral leaflet (in TMVR, valve-in-ring procedures, and mitral annular calcification) or from the deflected bioprosthetic leaflets and stent frame (in valve-in-valve procedures). Post-processing for neo-LVOT prediction typically involves the following: (1) segmentation of the mitral annulus or sewing ring; (2) definition of the annular trajectory, which is orthogonal to the annular plane and usually distinct from the LV long axis; (3) simulation of the transcatheter valve by superimposing a device model, aligned with the annular trajectory, with adjustable implantation depth; segmentation of the anticipated neo-LVOT using a centerline-based approach; and (4) planimetric measurement on cross-sectional reformats perpendicular to the neo-LVOT centerline. Neo-LVOT narrowing is promoted by increased ventricular protrusion of the device; flaring toward the LV outflow; larger aorto-mitral angulation; and more pronounced septal hypertrophy or bulging (9) (Figures 1, 2).

### Transseptal puncture: planning and selection of the site

In percutaneous mitral valve implantation procedures, careful planning of the transseptal puncture site is critical to achieve optimal device coaxiality and controlled valve deployment. Pre-procedural imaging, particularly CT and transesophageal echocardiography, is used to identify a posterior and inferior puncture location on the interatrial septum, providing an adequate height above the mitral annular plane and a favorable trajectory toward the center of the mitral valve. Selection of this puncture site improves alignment of the delivery system with the mitral annulus, reduces excessive catheter angulation, and enhances procedural stability, thereby minimizing the risk of malposition, paravalvular leak, and interference with surrounding cardiac structures.

### Transapical access and its possible complications

The TA approach, although providing direct coaxial access for TMVI, is associated with higher early complication rates compared with transseptal access. Apical access complications occur in ∼6% of cases and are strongly associated with increased requirements for circulatory support, higher rates of surgical conversion, and elevated early mortality. Meta-analytic data confirm higher 30-day mortality (14% vs. 4.7%) and major bleeding (19% vs. 10%) with TA access. Additional risks include LV perforation (<1%), circumflex artery injury, and LVOT obstruction (∼1%). Infectious events—mainly apical wound infections—occur in 1%–3% of patients and may rarely progress to deep thoracic infection or mediastinitis (<1%) (4, 10).

### Procedural technique

All procedures were performed in a catheter laboratory room under general anesthesia with full hemodynamic monitoring and real-time transesophageal echocardiographic (TEE) and fluoroscopic guidance.

**Figure 3:**
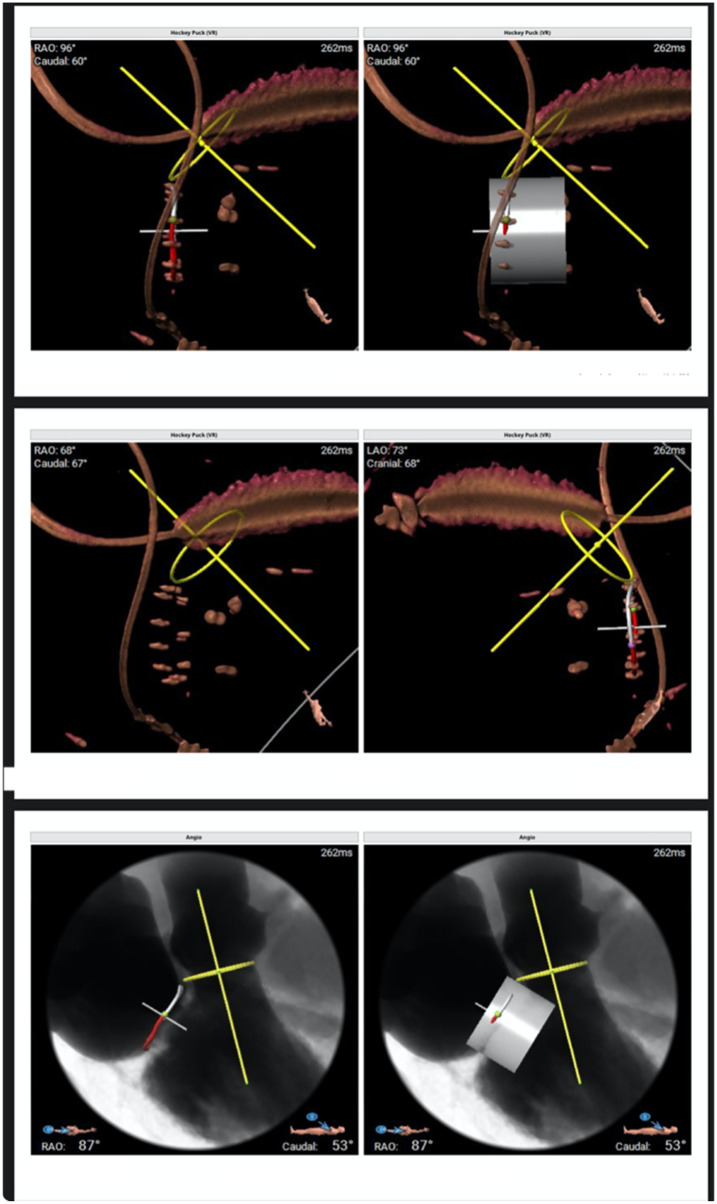
Three-dimensional volume-rendered reconstructions and multiplanar reformations are used to define the optimal fluoroscopic projections (“hockey puck” views) and to assess device coaxiality relative to the mitral annular plane.

**Figure 4:**
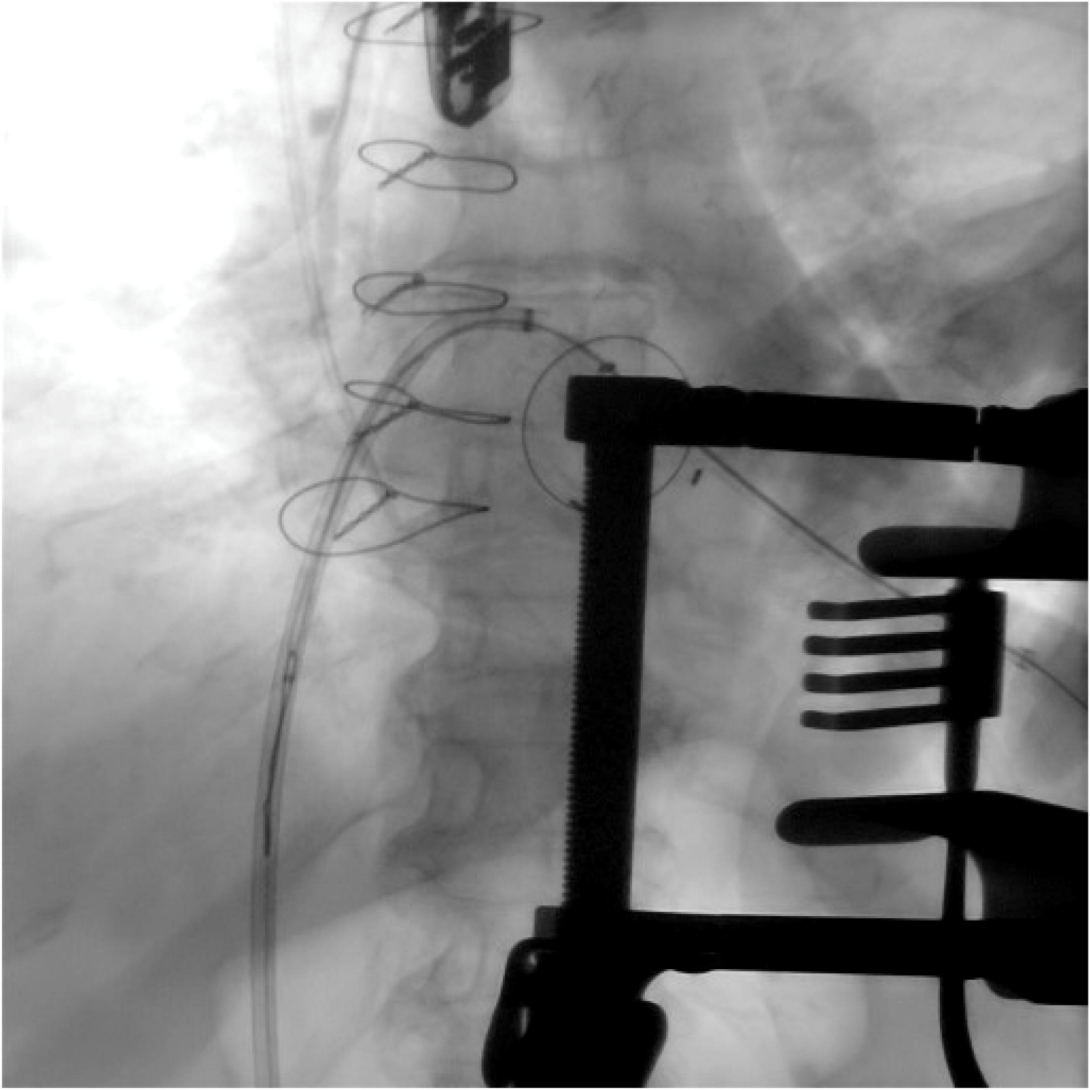
The snaring via the transseptal catheter of a soft-tipped guidewire introduced through a small-gauge apical needle into the LV cavity, passed across the degenerated mitral prosthesis into the left atrium.

**Figure 5:**
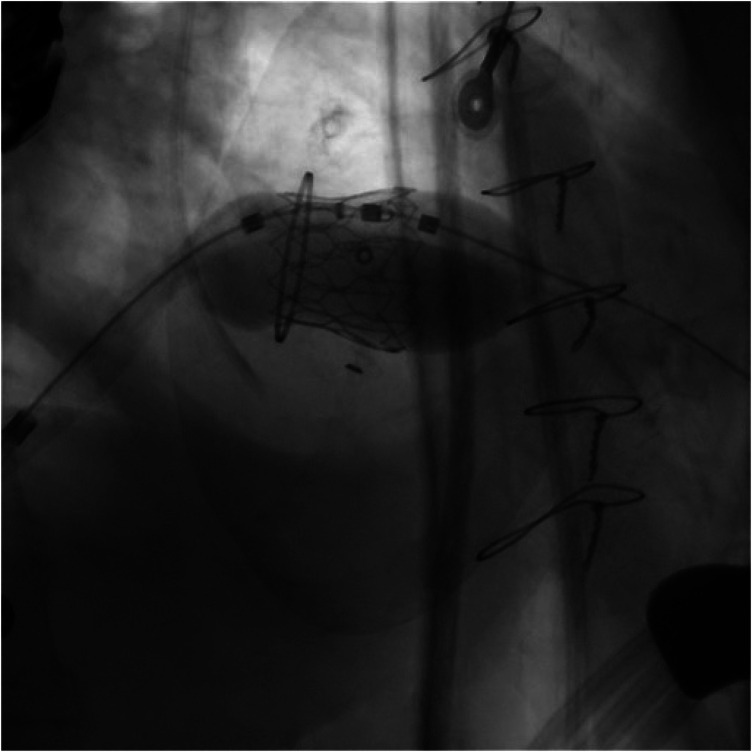
The deployment of the balloon-expandable valve.

**Figure 6:**
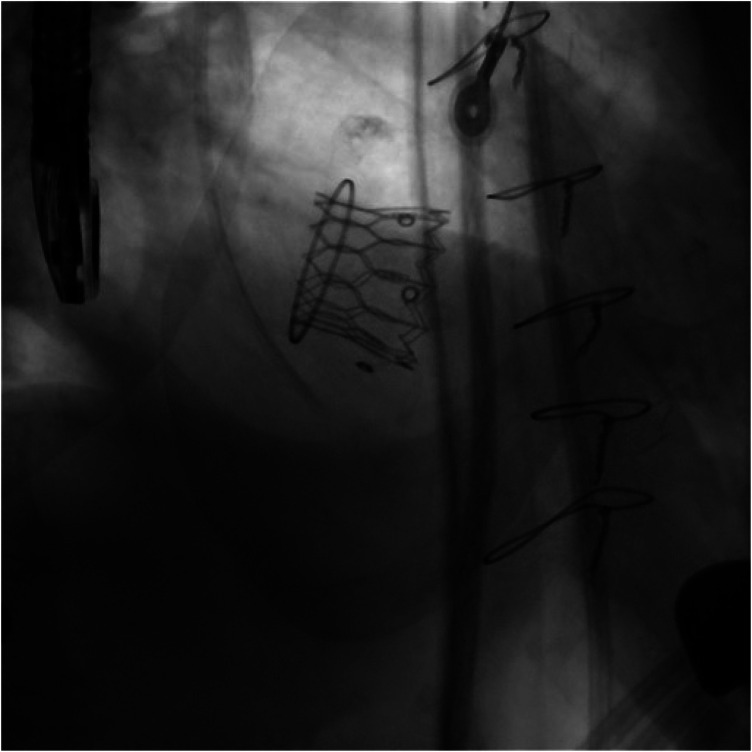
The final fluoroscopy result of the new implanted valve (ViV TMVI).

The cable-assisted TMVI technique followed a standardized protocol (Figure 3):

Right femoral venous access was obtained under ultrasound guidance, with pre-closure of this access using two Perclose-Proglide devices (Abbott, United States).
Ultrasound-guided (TEE) transseptal puncture was performed using a BRK transseptal needle and Fast Cath 8 Fr introducer (Abbott, United States).
A left mini-thoracotomy was performed in the fifth or sixth intercostal space, and the left ventricular apex was exposed without cardiopulmonary bypass.
A soft-tipped guidewire 0.035” (Merit Medical, United States) was introduced through a small-gauge apical needle into the LV cavity, passed across the degenerated mitral prosthesis or MAC into the LA, and snared via the transseptal catheter using an Amplatz Goose Neck snare kit (Metdtronic, United States) (Figure 4).
The wire was externalized through the femoral vein, establishing a through-and-through rail from femoral access to the apex. No introducer or large sheath was inserted through the apical site.
The externalized wire was exchanged for an Amplatz SuperStiff 0.035″ wire (Boston Scientific, United States).
Septostomy was performed by inflation using an EverCross PTA Balloon Catheter (Medtronic, United States).
The Myval THV was crimped and advanced entirely via the femoral route, with the transapical rail providing counter-traction and coaxial stability.
Deployment of the balloon-expandable valve was performed under combined fluoroscopic and TEE guidance, confirming optimal alignment, full expansion, absence of PVL, and freedom from LVOT obstruction (Figures 5, 6).
The apical access site was closed using pledgeted purse-string sutures, with direct hemostasis confirmed visually and by TEE. The right femoral venous access was closed using the two pre-entered Perclose-Proglide devices (Abbot, United States).

No patient required mechanical circulatory support, rapid pacing, or surgical conversion.

### Study population

Three patients (two men, one woman) underwent the procedure during the study period. The mean age was 71.7 years (range 65–78) (Table 1). All patients were in New York Heart Association (NYHA) functional class III or IV preoperatively. Two patients had a preserved left ventricular ejection fraction (≥55%), while one patient had a severely reduced ejection fraction (35%). Two patients had prior mitral bioprostheses, and one presented with severe MAC. Comorbidities included atrial fibrillation, renal impairment, chronic wound infection, and pleural effusion.

**Table 1:** Summary of the fundamental information of the three patients.

Patient	Age	Sex	Mitral pathology	Myval type	LVEF (%)	NYHA class	Comorbidities
1	65	M	Severe MAC with MS	Octacor 32 mm	60	IV	Chronic wound infection, renal impairment
2	78	F	Degenerated bioprosthesis (Hancock II 27), severe MR	Octapro 24.5 mm	55	III	AF, pleural effusion
3	72	M	Degenerated prosthesis (Medtronic 27) with combined MS/MR Malposition of the new implanted prosthesis	Octapro 26 mm	35	III	Dilated cardiomyopathy with reduced ejection fraction, renal impairment, AF

LVEF, left ventricular ejection fraction; MAC, mitral annular calcification; MS, mitral stenosis; MR, mitral regurgitation.

### Data collection and endpoints

All procedural and clinical data were collected prospectively in a dedicated institutional database. Outcome measures included the following:

Technical success, defined as successful implantation of the THV in the intended position without embolization, need for surgical bailout, or significant PVL.
Hemodynamic performance, assessed via post-deployment TEE, including mean transvalvular gradient, PVL grade, and LVOT patency.
Peri-procedural complications, including vascular injury, apical bleeding, arrhythmias, stroke, or access-site complications.
Clinical status at discharge, including NYHA functional class and need for readmission or escalation of care.

All patients underwent repeat transthoracic echocardiography prior to discharge and were scheduled for standard outpatient follow-up.

The study was approved by the Institutional Review Board (IRB No. PVE MOD 2025-04) and complied with the Declaration of Helsinki. Written informed consent for surgical treatment, data collection, and follow up was obtained from all patients.

## Results

### Procedural feasibility and patient demographics

Between October 2024 and May 2025, three high-risk patients underwent transcatheter mitral valve implantation (TMVI) utilizing a cable-assisted hybrid approach with the Myval transcatheter heart valve. All procedures were successfully completed without the need for surgical conversion or cardiopulmonary bypass, demonstrating the procedural feasibility and reproducibility of this novel technique.

The study cohort comprised two men and one woman, with a mean age of 71.7 years (range 65–78 years). All patients presented with severe symptomatic mitral valve disease and were classified as NYHA functional class III or IV at baseline. Two patients had previously undergone surgical mitral valve replacement with bioprosthetic degeneration, while the third presented with severe mitral annular calcification (MAC) and restrictive leaflet mobility. In two cases, the left ventricular ejection fraction (LVEF) was preserved (≥55%), while in one case, it was severely reduced (35%).

Relevant comorbidities included atrial fibrillation, moderate pleural effusion, chronic wound infection, dilated cardiomyopathy, and renal dysfunction. Each patient was deemed inoperable or at prohibitive surgical risk by a multidisciplinary heart team, based on EuroSCORE II and global clinical evaluation. Comprehensive pre-procedural imaging with transthoracic and transesophageal echocardiography, as well as contrast-enhanced cardiac CT, confirmed anatomical indications and procedural eligibility.

### Technical execution and device delivery

The hybrid cable-assisted TMVI technique was executed according to a standardized protocol in all patients. A stable through-and-through guidewire rail was established by externalizing a guidewire from the right femoral vein through the left atrium and left ventricle, and out via a surgically exposed apical puncture site. This rail provided a tensioned axis of stability that permitted precise delivery of the transcatheter valve system exclusively through the femoral route. Importantly, the apical site was not used for prosthetic valve advancement or deployment, thus preserving myocardial integrity and minimizing apical trauma.

All three Myval devices were successfully implanted under real-time transesophageal echocardiography (TEE) and fluoroscopic guidance, with appropriate coaxial alignment and controlled expansion achieved in each case.

### Patient-level outcomes

**Figure 7:**
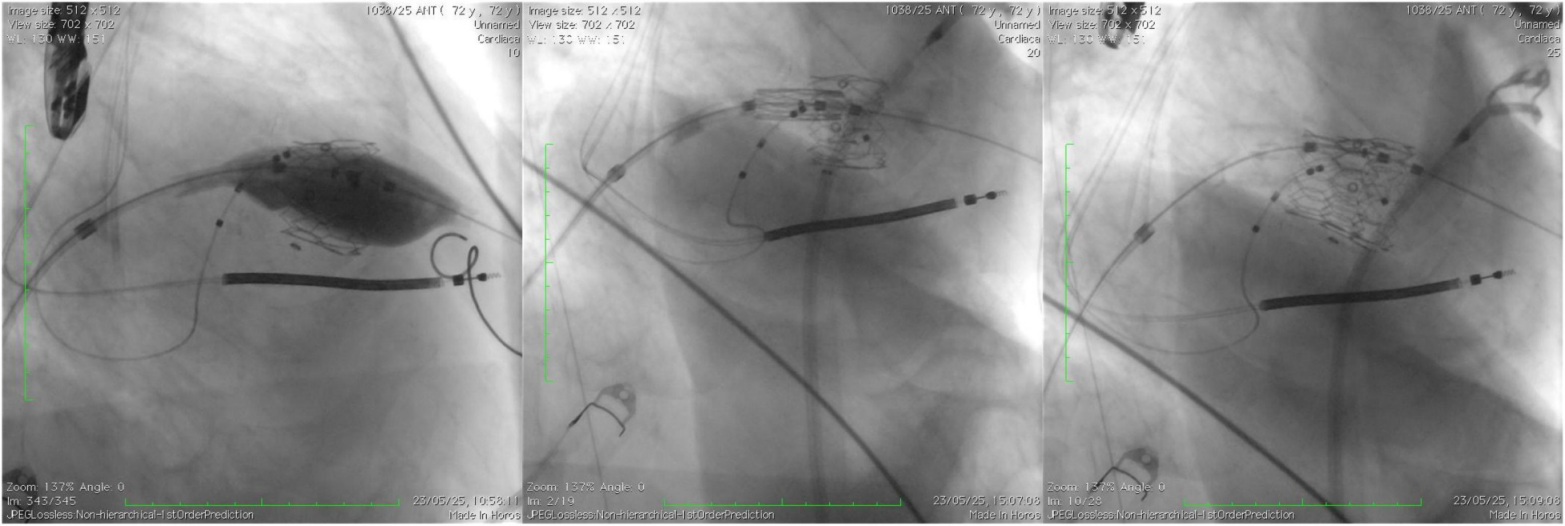
A comparative image illustrating the initial valve implantation, highlighting the pop-out occurring during the final release phase, as well as the positioning and deployment of the second valve following the creation of the rail technique (case 3).

Patient 1: A 65-year-old man with severe MAC (Mitral Annular Calcification) and symptomatic mitral stenosis received a 32-mm Myval Octacor valve. The procedure was technically straightforward, with a post-deployment gradient of 4.5 mmHg and only trace paravalvular regurgitation. No left ventricular outflow tract (LVOT) obstruction occurred. The patient experienced a localized wound infection at the thoracotomy site, successfully managed with vacuum-assisted closure (VAC) therapy. He did not develop valve-related complications and was discharged in NYHA class II.
Patient 2: A 78-year-old woman with a degenerated 27-mm Hancock II surgical bioprosthesis and severe mitral regurgitation underwent successful implantation of a Myval Octapro 24.5-mm valve. Procedural imaging confirmed excellent coaxiality and valve expansion. The final transvalvular gradient was 5.8 mmHg, with no evidence of paravalvular leak. Postoperatively, the patient developed transient atrial fibrillation and mild pleural effusion, both managed conservatively. She was discharged in NYHA class II, with preserved LVEF and no signs of device dysfunction.
Patient 3: A 72-year-old man with a failed mitral bioprosthesis and mixed mitral pathology initially exhibited malalignment and early migration of the valve upon deployment. However, the cable-assisted rail provided sufficient stability to allow for controlled repositioning without hemodynamic compromise. A second Myval Octapro 26-mm valve was successfully implanted in the correct position. Post-procedural echocardiography demonstrated normal valve function, no residual mitral gradient, and complete absence of paravalvular leak. The patient remained hemodynamically stable throughout hospitalization (Figure 7).

### Hemodynamic and clinical outcomes

Across all three patients, technical success—defined as accurate valve deployment in the intended position without embolization, significant paravalvular leak, or need for conversion to surgery—was achieved in 100% of cases. No patient required rapid pacing, mechanical circulatory support, or extracorporeal assistance during or after the procedure.

There were no instances of peri-procedural stroke, access site complications, or LVOT obstruction. Echocardiographic assessments performed prior to discharge confirmed optimal prosthesis seating and acceptable mean transvalvular gradients (range 4.5–5.8 mmHg).

From a clinical standpoint, all three individuals demonstrated a marked improvement in functional status, transitioning from NYHA class III/IV to class I/II at the time of discharge. No readmissions or adverse cardiac events were reported during the initial hospitalization period.

## Discussion

TMVI has established itself as a life-saving option for inoperable or high-risk patients affected by degenerated mitral prostheses, annuloplasty failure, or severe mitral annular calcification. Despite these advances, the anatomical complexity of the mitral valve remains a significant challenge for standard transseptal access, particularly when optimal coaxial alignment and device stability are required.

In this context, hybrid access strategies have emerged as valuable intermediate solutions between fully percutaneous and surgical implantation. Among these, the cable-assisted approach—creating a through-and-through guidewire rail between femoral and transapical access points—has been proposed to improve control over device delivery. However, this technique remains scarcely explored in the current literature.

To date, no published case series has described the cable-assisted rail technique specifically in combination with the Myval valve. Although the Myval THV has already been used off-label in mitral settings with favorable outcomes (11–13), none of these experiences employed hybrid stabilization or transapical guidance strategies, underscoring the novelty of the present approach.

Although isolated reports using other platforms, such as the SAPIEN 3 (1, 3) or Tendyne valves (14), have mentioned rail-like or apically guided delivery strategies, these descriptions are limited to anecdotal, single-case experiences. In most of these, transapical access was used exclusively for direct device deployment, without the integration of a tensioned stabilizing rail extending through the vascular route. Moreover, no structured series to date has systematically explored the role of a cable-assisted rail technique specifically in anatomically complex scenarios—such as valve-in-ring or valve-in-MAC cases—using balloon-expandable transcatheter systems. These anatomical substrates are particularly prone to issues of valve instability, paravalvular leak, and malcoaptation due to poor coaxiality and challenging annular geometry.

Against this backdrop, our series represents the first documented experience combining a balloon-expandable platform—namely, the Myval THV—with a hybrid cable-assisted stabilization strategy. This novel approach provides enhanced alignment, anchoring support, and controlled deployment in high-risk anatomies, while avoiding the invasiveness of full transapical systems. Its feasibility and safety profile in this context may offer a new procedural alternative for operators facing suboptimal delivery conditions via conventional transseptal access.

While the Myval transcatheter heart valve was originally conceived for aortic implantation, several off-label reports have validated its feasibility in mitral valve-in-valve (ViV) and valve-in-ring (ViR) procedures. For instance, Blasco-Turrion et al. (11) described successful mitral ViV implantation using Myval, noting favorable hemodynamic outcomes and positioning, although via transfemoral-transseptal access. Similarly, Senguttuvan et al. (12) reported a complex case involving Myval TMVI combined with paravalvular leak closure, again without employing hybrid techniques. Sankardas et al. (13) analyzed Myval ViV TMVI in a larger Indian cohort, suggesting acceptable gradients and clinical improvement, yet exclusively through a transseptal route.

These studies confirm the hemodynamic compatibility and procedural feasibility of Myval in the mitral position. However, none have reported the use of transapical anchoring or cable-guided stabilization, which further underscores the novelty of our approach.

The cable-assisted configuration provides distinct procedural advantages. It enhances coaxiality, a critical factor in successful valve deployment. It stabilizes device delivery even in tortuous or heavily calcified anatomies, reduces the need for large-caliber introducers through the apex, and preserves full femoral delivery, maintaining a low-profile procedural footprint. In our series, one patient required intra-procedural repositioning due to suboptimal alignment. This event was managed effectively using the cable rail, without any hemodynamic instability. Ultimately, all three implants were successfully deployed, with no need for surgical conversion or extracorporeal support.

Clinically, all patients demonstrated improvement in NYHA functional class and echocardiographic findings. Post-procedural gradients ranged from 4.5 to 5.8 mmHg, with no occurrences of valve embolization, LVOT obstruction, or significant paravalvular leak. These results suggest that the cable-assisted method may represent a reproducible, safe, and anatomically adaptable alternative for TMVI in complex patients, particularly where standard transseptal access may be excessively risky.

To the best of our knowledge, this is the first reported series describing the cable-assisted technique for TMVI using the Myval valve. It fills a gap in the evolving spectrum of mitral transcatheter techniques, combining the benefits of hybrid access with the flexibility of a low-profile balloon-expandable system. As TMVI continues to expand into off-label and bailout scenarios, technical refinements such as the one described here could enhance procedural safety, reduce complications, and broaden eligibility in anatomically unfavorable cases.

## Conclusions

This technical report presents the first documented experience of TMVI using a cable-assisted hybrid delivery strategy with the Myval transcatheter heart valve. The approach proved feasible, safe, and reproducible in three high-risk patients with complex mitral anatomies, including degenerated surgical prostheses and severe mitral annular calcification.

The through-and-through rail technique enabled stable device navigation and coaxial alignment, while minimizing surgical trauma at the apical site. All procedures achieved technical success without the need for cardiopulmonary bypass or valve embolization, and were associated with favorable early hemodynamic and clinical outcomes.

Our experience supports the cable-assisted configuration as a valuable adjunct in the TMVI toolbox—particularly for anatomies that preclude safe transseptal deployment or demand enhanced prosthesis control. The use of the Myval THV in this setting adds flexibility due to its low-profile design, intermediate sizing, and balloon-expandable platform.

### Future directions

In light of the technical success and clinical safety demonstrated within this limited series, there is clear rationale for further evaluation of the cable-assisted TMVI approach. Expanding the investigation through larger prospective registries would allow for a more comprehensive assessment of its performance across a broader spectrum of anatomical profiles. Such studies should particularly focus on key aspects including long-term structural valve function, procedural reproducibility across different clinical centers, and potential applicability in specific scenarios, such as redo mitral surgery or cases requiring exclusion of mitral annular calcification.

Beyond procedural expansion, future developments may also benefit from the integration of advanced pre-procedural imaging techniques, such as CT-derived rail planning and computational flow modeling. The adoption of standardized valve sizing algorithms could further refine procedural planning and improve clinical outcomes.

In conclusion, our findings represent a foundational proof of concept, establishing hybrid access using the Myval system as a viable option within the TMVI landscape. This approach opens new possibilities for broader clinical adoption, particularly in anatomically complex or borderline cases where conventional strategies may present excessive risks.

## Data Availability

The raw data supporting the conclusions of this article will be made available by the authors, without undue reservation.
